# Cost-effectiveness of ALK testing and first-line crizotinib therapy for non-small-cell lung cancer in China

**DOI:** 10.1371/journal.pone.0205827

**Published:** 2018-10-23

**Authors:** Shun Lu, Yongfeng Yu, Shijun Fu, Hongye Ren

**Affiliations:** 1 Shanghai Lung Cancer Center, Shanghai Chest Hospital, Shanghai Jiao Tong University, Shanghai, China; 2 Pfizer China Medical Department, Pfizer China Inc., Beijing, China; CCAC, UNITED STATES

## Abstract

**Introduction:**

Anaplastic lymphoma kinase (ALK) rearrangement gene testing is used increasingly to identify patients with advanced non-small-cell lung cancer (NSCLC) who are most likely to benefit from crizotinib. This study was to evaluate the cost-effectiveness of the ALK tests followed by crizotinib compared to the standard chemotherapy in advanced NSCLC from the Chinese healthcare system perspective.

**Methods:**

A 10-year Markov model was constructed to compare the costs and quality-adjusted life-years (QALYs) of crizotinib with standard chemotherapy, guided by the ALK rearrangement tests: next-generation sequencing (NGS) panel tests and multiplex polymerase chain reaction (PCR) testing. The health states included progression-free survival (PFS), progressed survival, and death. The costs examined included cost of drugs (pemetrexed, standard chemotherapy, salvage chemotherapy, and crizotinib), follow-up, palliative care, supportive care, severe adverse events, and ALK rearrangement testing.

**Results:**

Under Patient Assistance Program (PAP), the model demonstrated that the patients using NGS panel tests spent US $31,388 and gained 0.780 QALYs, whereas patients using multiplex PCR spent US $31,362 and gained 0.780 QALYs, respectively. The incremental cost-effectiveness ratios of crizotinib with PAP compared to the control strategy were projected at $14,384 (NGS) and $13,740 (multiplex PCR) per QALY gained, respectively. Sensitivity analyses showed the utility of PFS and the costs of crizotinib and pemetrexed were the most impactful factors on the model outcomes. The results were robust to changes in all parameters.

**Conclusion:**

ALK-rearrangement test positive followed by crizotinib may be cost-effective compared to standard chemotherapy from the Chinese healthcare system perspective when PAP was available.

## Introduction

Lung cancer is the most commonly diagnosed cancer with an approximate 651,053 and 733,300 newly diagnosed cases, as well as the leading cause of cancer-related mortality in China, with 529,153 and 610,200 deaths from the disease in 2011 and 2015, respectively.[1, 2] The number of new diagnosis increased by 34.8% from 2005 to 2011.[3] Non-small-cell lung cancer (NSCLC) accounts for approximately 85% (range: 80% and 90%) of lung cancer,[4–8] approximately 40% of which are first diagnosed at the advanced stage of malignancy.[9] It was reported that the 1-year overall survival rate of NSCLC ranges from 30% to 46%,[10, 11] while the 5-year overall survival rate was estimated at 19%.[12] Studies show that lung cancer has a significant economic burden on the health care system, with an average annual cost of 3.799 billion Chinese Yuan in 2005.[3] Healthcare spending for NSCLC has increased significantly in the past in China, partially due to the newly-developed treatment options.[13]

Rearrangement of anaplastic lymphoma kinase (ALK) gene occurs in NSCLC.[14, 15] Crizotinib, an oral small-molecule tyrosine kinase inhibitor (TKI) of ALK, dramatically increases objective response rates (RR) and progression-free survival (PFS) in patients diagnosed with advanced NSCLC with ALK rearrangements compared with standard chemotherapy as first-line treatment (RR, 75% and 45%, p<0.001; median PFS, 10.9 months vs. 7.0 months; hazard ratio [HR], 0.45, 95% confidence interval [CI], 0.35 to 0.60; p<0.001).[16] Crizotinib was approved as the first-line treatment for advanced ALK-positive NSCLC in many countries, including the United States, the European Union (EU), and China.

Except ALK, EGFR and ROS1 are also commonly tested biomarkers in clinical practice. The next-generation sequencing (NGS) panel testing and multiplex polymerase chain reaction (PCR) based assay are the two most commonly used lab testing methods to detect all these "druggable" targets simultaneously. The NGS panel testing offer opportunities to obtain additional data on lung cancer molecular mechanisms and targeted oncogenic pathways. The NGS panels may be largely different from different companies. It causes different test costs using different NGS panels. However, the multiplex PCR is sometimes used with more consistent price compared to NGS panel tests. Treatment for NSCLC with crizotinib results in substantially higher drug expenses because of high price of crizotinib. Limiting this treatment to patients with ALK rearrangement-positive tumors is one potential way to improve the economic outcome of crizotinib maintenance treatment. Therefore, it is important to evaluate the cost-effectiveness of the treatment of advanced NSCLC with ALK rearrangement, identified by available testing.

Recent study shown that ALK rearrangement positive patients receiving crizotinib treatment might be a cost-effective treatment strategy comparing with the traditional standard chemotherapy in the presence of the patient assistance program (PAP). Among the three genes testing followed by crizotinib therapy, patients with Ventana immunohistochemistry (IHC) had the highest incremental cost per quality-adjusted life-year (QALY) gained, followed by IHC testing plus fluorescent *in situ* hybridization (FISH) confirmation and quantitative reverse transcription-PCR (qRT-PCR) testing.[17] However, it is unknown whether or not the two genes testing, NGS panel tests and multiplex PCR testing, perform better than standard chemotherapy in terms of the cost-effectiveness in China. The aim of this study was to evaluate the cost-effectiveness of the two genes-guided testing followed by crizotinib treatment for advanced NSCLC compared to standard chemotherapy based on Chinese clinical and cost data.

## Methods

### Model description

A 10-year decision model was developed using the R statistical environment (version 3.2.2; R Development Core Team, Vienna, Austria) to evaluate the costs and QALYs associated with using two ALK rearrangement tests prior to crizotinib treatment strategies: NGS panel tests and multiplex PCR testing. Patients who were tested ALK-rearrangement positive would receive crizotinib treatment, while ALK-rearrangement negative would receive a standard chemotherapy.

The model was created with a hypothetical cohort of confirmed stage IIIB or IV NSCLC patients to compare the two ALK rearrangement tests guided treatment strategies with the control group (standard chemotherapy). Please see our previous study (Lu et al., 2016) for the model structure and assumptions. In addition, during each Markov cycle, the patients may remain progression-free or progressed. Once patients had a progressed disease, they remain in the progressed disease state until they die from NSCLC. Once a patient enters a health state, the patient will not progress to a better health state. After the cancer progressed, patients were treated with second-line chemotherapy or supportive care. The study was conducted from a Chinese healthcare system perspective. Costs and QALYs were discounted at an annual rate of 5% in the base case.[18]

The sensitivity and specificity of NGS panel tests were assumed to be 100%,[19] with a confidence interval (CI) of 0.95 to 1. The sensitivity and specificity of the multiplex PCR testing were obtained from a published literature (Marchetti et al., 2016). (Table 1)

**Table 1:** Key clinical data.

**Parameter**	**Values (ranges)**	**Description and Reference**
**Weibull survival model of PFS of crizotinib in Chinese cohort**	Scale = 0.0211; Shape = 1.5326; r^2^ = 0.972	Solomon et al., 2014
**Weibull survival model of PFS of PC in Chinese cohort**	Scale = 0.0663; Shape = 0.8604; r^2^ = 0.991	Solomon et al., 2014
**HR of survival of chemotherapy versus supportive care**	0.77(0.83–0.71)	NSCLC Meta-Analyses Collaborative Group, 2008
**Probability of progressed survival**	0.086(0.08–0.093)	Hayashi et al., 2012
**ALK prevalence**	0.065(0.014–0.116)	Wu et al., 2013
**Body surface (m^2^)**	1.72(1.5–1.9)	Wu et al., 2011
**NGS panel tests**		
**Sensitivity**	1(0.95–1)	Lin et al., 2016
**Specificity**	1(0.95–1)	Lin et al., 2016
**Multiplex PCR testing**		
**Sensitivity**	1(0.95–1)	Marchetti et al., 2016
**Specificity**	1(0.95–1)	Marchetti et al., 2016

ALK: anaplastic lymphoma kinase; HR: hazard ratio; NGS: next-generation sequencing; NSCLC: non-small-cell lung cancer

PC: pemetrexed plus cisplatin; PFS: progression-free survival; PCR: polymerase chain reaction

### Clinical data

Model inputs for clinical data were obtained from the published randomized clinical studies or meta-analyses (Table 1). The Kaplan-Meier survival curve for PFS of crizotinib and standard chemotherapy were obtained from the Phase 3 Study of First-Line Crizotinib vs Pemetrexed−Cisplatin/Carboplatin (PC) in East Asian Patients with ALK+ Advanced Non-Squamous Non-Small Cell Lung Cancer (PROFILE 1029) study.[20]

Weibull survival model was fitted to the reported PFS of crizotinib or PC in Chinese cohort. The estimated scale, shape (Ƴ), and adjusted r-square of Weibull survival models and other clinical data are presented in Table 1.

It was assumed that patients had an average weight of 65 kg and height of 164 cm, resulting in a body surface area of 1.72 m^2^. The area of body surface was used to calculate the chemotherapy agent’s dosage and cost. It was assumed that crizotinib 250 mg twice daily was administered to the ALK rearrangement test-positive patients until disease progression, while chemotherapy was administered 21 days per cycle for four consecutive cycles. It was assumed that the patients’ PFS with a false-positive ALK rearrangement test result followed by crizotinib treatment were the same as those who received supportive care. Following disease progression, salvage chemotherapy and supportive care would be provided, with 56.6% (26%–72%) of patients would receive salvage treatment regardless of the first-line therapy.[21–26] The resource utilization related to salvage chemotherapies and supportive care was derived from a previously published study.[27]

The HR of the overall survival for chemotherapy versus supportive care was obtained from the published systematic literature review.[28] The probability of progressed survival (PS) was derived from a study, with an identification of 69 clinical trials of first-line chemotherapy in advanced NSCLC and an indication of median overall survival as 5.4 months after disease progression.[29]

### Costs

Unit costs were obtained from various sources. All costs are reported in 2016 US dollars. Costs from a different base year were not inflated given that the Chinese healthcare costs are stable under the central control by the government. Direct medical costs were included in the model, including the ALK rearrangement test cost, first- and second-line chemotherapy cost (consisting of prescription, preparation, administration, and hospitalization cost), concomitant medication cost, management of treatment-related serious adverse events (SAEs) cost, and routine follow-up and lab tests cost (Table 2). The cost of ALK rearrangement test per patient was obtained from Chinese hospital laboratories.

**Table 2:** Cost input.

**Costs (US dollars) Parameter**	**Base-case value**	**Range**	**Description**	**Reference**
**Cost of pemetrexed per 500 mg**	2083.97	857.14–2126.51	Local charge	
**Cost of traditional chemotherapy per cycle other than pemetrexed**	518.4	388.8–648	Local charge	
**Cost of crizotinib per day**	238.1	119.05–238.1*	Local charge	
**Cost of follow-up per unit**	55.6	41.7–69.4		Wu et al., 2014
**Cost of salvage chemotherapy per cycle**	2352.7	1921.1–4383.3		Wu et al., 2014
**Cost of palliative care in end-of-life**	2042.91	793.65–5456.19		Lu et al., 2016(2)
**Cost of supportive care per cycle**	337.5	158.7–793.7		Wu et al., 2014
**Cost of SAEs in initial chemotherapy per cycle**	507.4	189.7–825.0		Wu et al., 2014
**Cost of ALK rearrangement testing**				
**NGS**	1,014.49	869.57–1,159.42	Local charge	
**Multiplex PCR**	660.75	440.50–881.01	Local charge	

* The half of expense of crizotinib was assumed for the one-way sensitivity analysis.

NGS: next-generation sequencing; PCR: polymerase chain reaction

Cost of palliative care at the end of life and the cost of SAEs in initial chemotherapy per cycle was obtained from a published study[17] (Table 2). The analyses were conducted with and without the presence of crizotinib patient assistance program (PAP), respectively (Lu et al., 2016).

The Chinese government has constructed a specific insurance system to provide up to approximately $32,000 US dollars per year for patients with a catastrophic disease such as advanced cancer.[17] Due to the lack of a specific willingness-to-pay (WTP) threshold for Chinese patients with cancer, the current analysis used $32,000 US dollars per QALY gained as the threshold of cost-effectiveness.

### Utility

**Fig 1:**
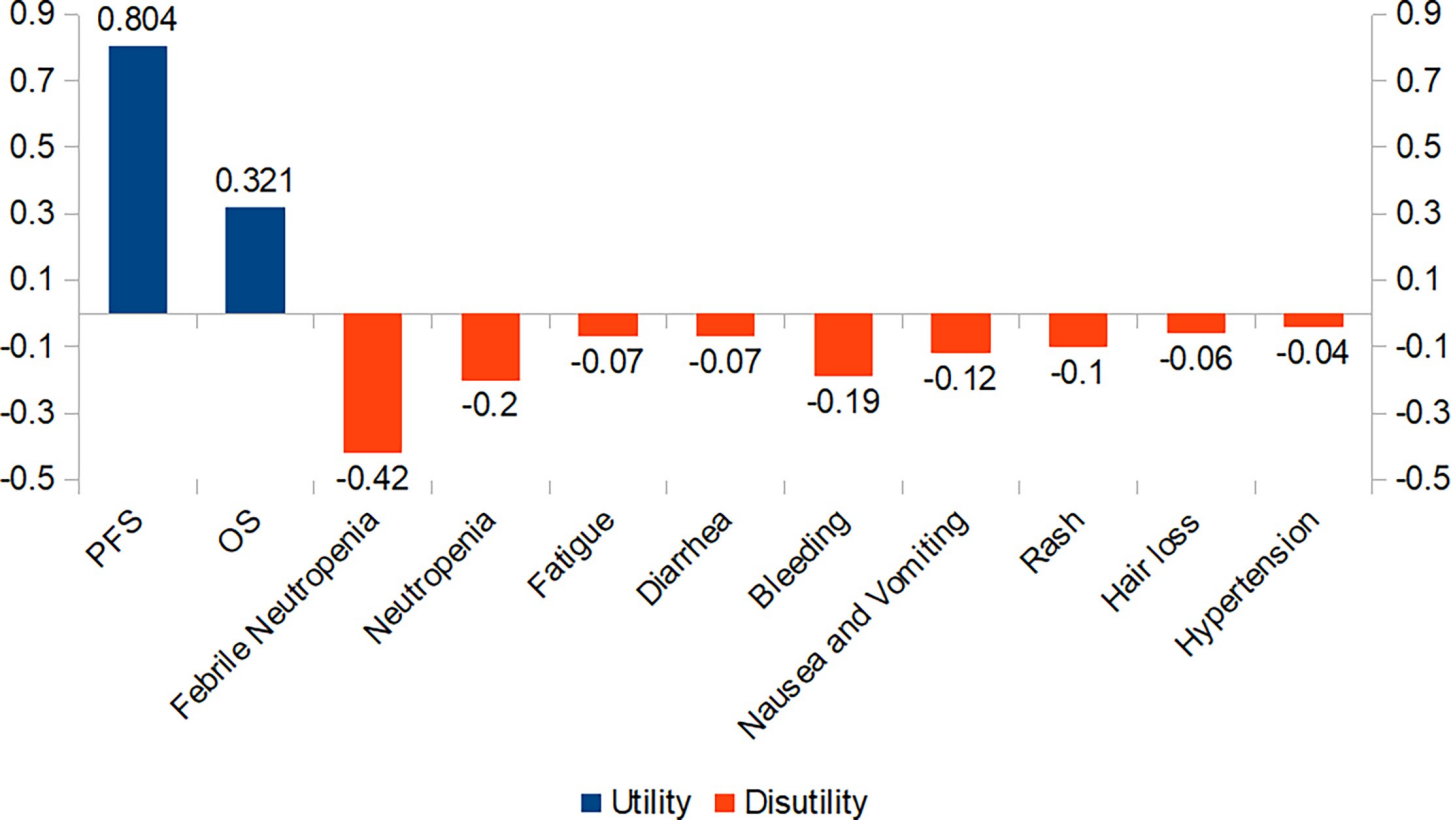
Health utilities and disutilities of advanced NSCLC. NSCLC: non-small-cell lung cancer; PFS: progression-free survival; OS: overall survival.

The utility scores of PFS and OS were obtained from published studies, which were 0.804 and 0.321, respectively (Fig 1), with a 25% range of the mean (±SE) in sensitivity analysis.[30, 31] The reported disutility caused by SAEs was used in the model, including febrile neutropenia, neutropenia, fatigue, diarrhea, bleeding, nausea and vomiting, rash, hair loss, and hypertension.[32–34]

### Outcomes

This is one model study, all data used in the model is from public available data, and cited in the reference and mentioned in previous method. [18, 20–31] The model generated estimates of the health care costs, QALYs, incremental cost per QALY gained, incremental cost-effectiveness ratio (ICER), disease-free life years (LYs), and overall LYs. The ICER estimated the differences in cumulative costs divided by the differences in QALYs between the ALK rearrangement tests guided treatment strategy and standard chemotherapy without gene screening. All the outcomes were presented when the PAP was available and when the PAP was not available.

### Sensitivity analyses

One-way sensitivity analyses were performed on all parameters at *a priori* defined ranges shown in Tables 1 and 2, while holding the other variables fixed to assess the robustness of the Markov model. All the ranges were mainly obtained from published studies, clinical trials, or with an assumption of 25%-50% range of the base-case value.

A Monte Carlo simulation probabilistic sensitivity analysis (PSA) was carried out to determine the effect of uncertainty in the key model parameters on the cost and QALY for each strategy. We conducted the Monte Carlo simulation from the set parametric distributions to ensure the reliability of the model. Triangle distributions were utilized for cost parameters other than crizotinib cost, while beta distributions were selected for parameters including probability, proportion, and preference value. A cost-effectiveness acceptability curve (CEAC) and the scatter plots of Monte Carlo simulation were developed based on the PSA results.

## Results

### Base-case analysis

The model shows that patients with ALK rearrangement followed by crizotinib treatment resulted in more health benefits and increased costs than control strategy (Table 3). When the PAP was available, patients whom received NGS panel testing to guide treatment spent US $31,388 and gained 0.780 QALYs, whereas patients whom received multiplex PCR testing to guide treatment spent US $31,362 and gained 0.780 QALYs, respectively. Compared to the control strategy, the ICERs of NGS and multiplex PCR screening followed by crizotinib were US $14,384 and US $13,740 per QALY gained, respectively. The results suggest that at a WTP threshold of $32,000 per QALY, the multiplex PCR testing and NGS panel testing are both acceptable, although multiplex PCR is a bit more cost-effective than NGS panel testing. The ICERs of the crizotinib treatment without PAP were projected at greater than US $174,000 per QALY gained compared to the control strategy.

**Table 3:** Summary of cost (US dollars) and outcome results in the base-case analysis.

**Strategy**	**Cost**	**Progression-free LYs**	**Overall LYs**	**QALYs**	Incremental cost per QALY*
**PC (control strategy)**	30,811	0.536	1.394	0.740	
**Crizotinib with PAP**					
**NGS panel tests**	31,388	0.581	1.450	0.780	14,384
**Multiplex PCR testing**	31,362	0.581	1.450	0.780	13,740
**Crizotinib without PAP**					
**NGS panel tests**	37,826	0.581	1.450	0.780	174,970
**Multiplex PCR testing**	37,800	0.581	1.450	0.780	174,327

* Comparing with Control strategy

LYs: life years; NGS: next-generation sequencing; PAP: patient assistance program; PC: pemetrexed plus cisplatin; QALY: quality-adjusted life-year; PCR: polymerase chain reaction

### Sensitivity analyses

**Fig 2:**
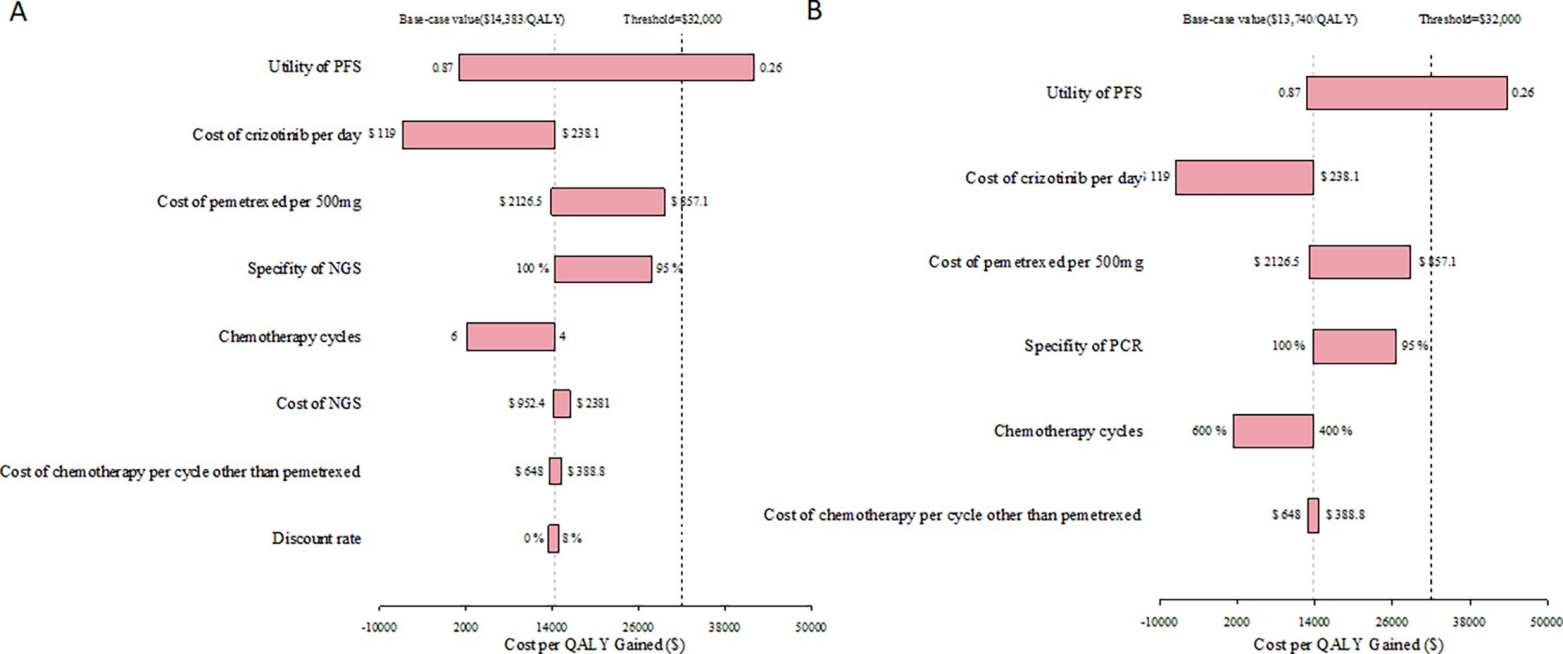
One-way sensitivity analyses for NGS panel tests and multiplex PCR versus control (with PAP). (A) NGS vs. control; (B) multiplex PCR vs. control. NGS: next-generation sequencing; PAP: patient assistance program; QALY: quality-adjusted life-year.

One-way sensitivity analyses were performed by varying the values of utilities, costs, and probabilistic parameters (Fig 2). The one-way sensitivity analyses showed that compared to the control strategy, the NGS panel testing and multiplex PCR testing followed by crizotinib treatment when PAP was available, the most sensitive parameters included: the utility of PFS, the cost of crizotinib per day, the cost of pemetrexed per 500 mg dose, and the specificity of NGS and multiplex PCR.

**Fig 3:**
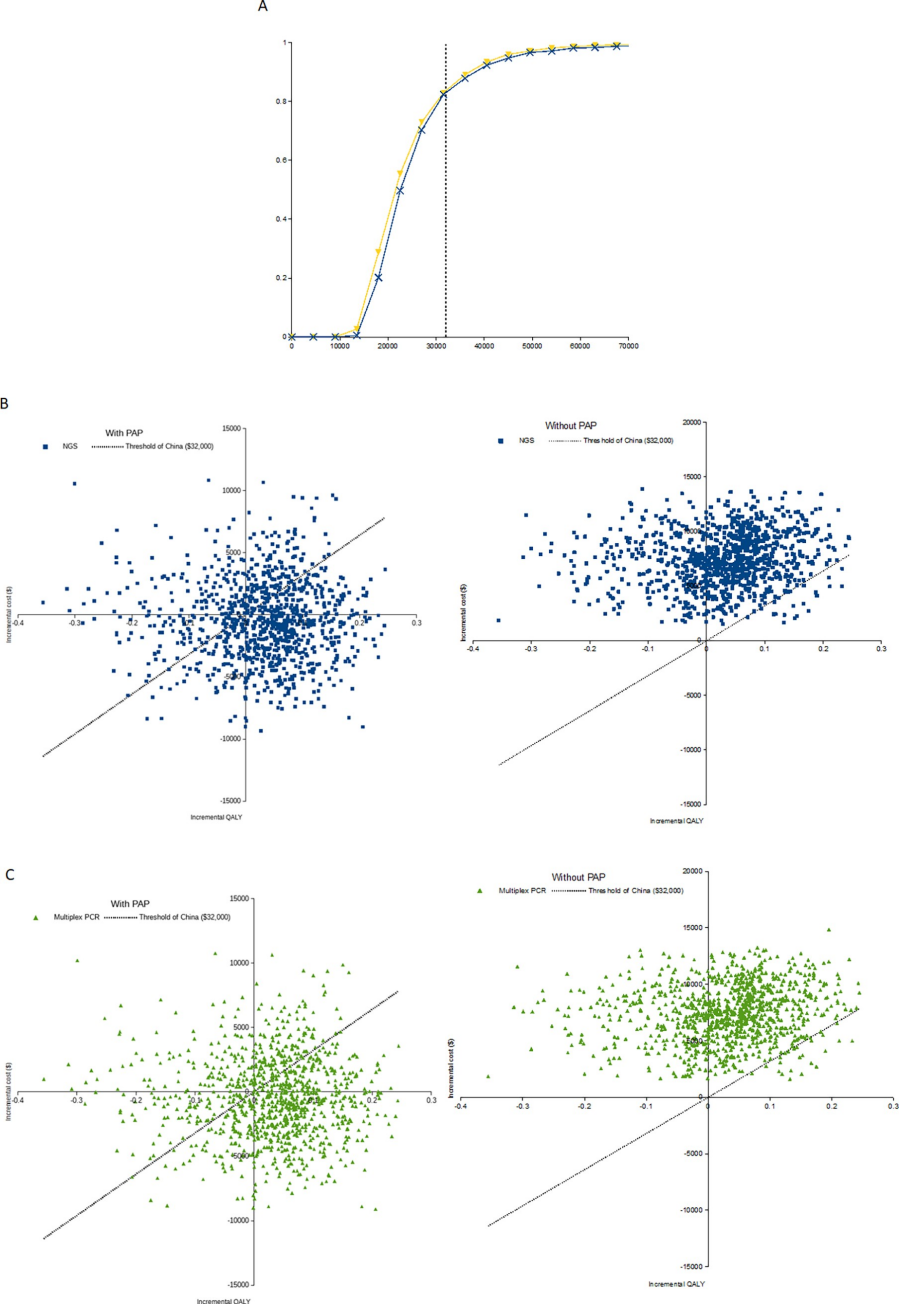
Probabilistic sensitivity analysis (PSA) results. (A) Cost-Effectiveness Acceptability Curves Comparing the Cost-effectiveness of Crizotinib Treatment with the ALK Rearrangement Testing (NGS and multiplex PCR) Versus Standard Chemotherapy, with PAP; (B) Scatter Plot of Monte Carlo Probabilistic Sensitivity Analysis, NGS vs. Control Strategy, with Patient Assistance Program (PAP); (C) Scatter Plot of Monte Carlo Probabilistic Sensitivity Analysis, Multiplex PCR vs. Control Strategy, with Patient Assistance Program (PAP). Blue line indicates NGS and yellow one is multiplex PCR, ALK: anaplastic lymphoma kinase; NGS: next-generation sequencing; PAP: patient assistance program; QALY: quality-adjusted life-year; PCR: polymerase chain reaction.

The PSA results were shown using the cost-effectiveness acceptability curves (CEAC) as well as the scatter plot of Monte Carlo simulation (Fig 3). We ran 1,000 simulations for the probabilistic sensitivity analysis. The CEAC curves show the percentage of simulations that favor one strategy (i.e., NGS and multiplex PCR, respectively) over the control strategy. When the PAP was available, compared to the control strategy with a cost-effectiveness threshold of US $32,000, the proportions of simulations with cost-effectiveness for NGS panel testing and multiplex PCR testing followed by crizotinib treatment for ALK rearrangement-positive NSCLC were 82.3% and 82.9%, respectively.

When the PAP was not available, the control strategy achieved 100% likelihood of cost-effectiveness and the likelihood of the two ALK rearrangement tests-guided treatment strategies were zero.

Fig 3B and 3C shows the scatter plot of Monte Carlo simulation. Those points below the WTP line mean cost-effective: the areas between the WTP line and x-axis in quadrant I (northeast), the areas between the WTP line and y-axis in quadrant III (southwest), and the areas of quadrant IV (southeast) (dominant, i.e., less costly and more QALYs).

## Discussion

We used a decision model to estimate the costs, QALYs, and incremental cost-effectiveness ratios of two commonly used ALK rearrangement screening tests, NGS panel tests and multiplex PCR testing, which are used to guide treatment decisions about crizotinib treatment in advanced NSCLC. These gene-guided treatment strategies were compared to the standard chemotherapy, considering the availability of the PAP.

When the PAP was available, the base-case analysis and the sensitivity analysis showed that ALK rearrangement test positive followed by subsequent crizotinib treatment resulted in an incremental cost per QALY gained of US $13,740 and US $14,384 for multiplex PCR and NGS, respectively. In the Chinese healthcare system setting, ALK rearrangement patients receiving crizotinib treatment may be cost-effective treatment strategies compared with standard chemotherapy treatment, when the PAP was available. Amongst the two ALK rearrangement testing methods, the multiplex PCR testing was a little more optimal than NGS panel testing, which might be due to the lower cost of the multiplex PCR testing. When the PAP was not available, the gene-guided treatment strategies may no longer be a cost-effective option compared to the standard chemotherapy given that the ICERs was approximately 6 times higher than the threshold of willingness-to-pay of $32,000 per QALY gained.

The new genomic diagnostics and personalized medicine applications are increasingly entering the market, especially for NGS and multiplex PCR. ALK, EGFR, ROS1 testing drive the application of new technologies in clinical practice and will shape the diagnostic panel deeply in Chinese market. The cost-effectiveness of various diagnostic methods will be keeping changing in the rapid evolution, with more approved target therapies in the future. Considering the increasing costs of targeted cancer treatment as well as gene diagnostic tests in oncology, our current study provides a valuable contribution to the literature and to policy makers. Our analysis may assist payers and health care providers in selecting the optimal gene-guided treatment strategy for advanced non-squamous NSCLC patients.

This study has several limitations. Like all mathematical models, our cost-effectiveness analysis relies on key assumptions. The available data for inputs into our model were limited; therefore, we made a few assumptions based upon a recently published study.[17] For example, according to the PROFILE 1029 study[20] which indicated that the overall survival was not significantly different between crizotinib treatment and standard chemotherapy, it was assumed that the risks of death were similar. Second, due to the lack of head-to-head comparison from clinical trial data, this current study was limited to invest the standard chemotherapy using cisplatin plus pemetrexed without other chemotherapy regimens. Given the favorable safety profile of cisplatin plus pemetrexed, it is acceptable being selected as control strategy in terms of the standard chemotherapy.[23] Further cost-effectiveness analyses are encouraged to be conducted when head-to-head comparison clinical trial data are available. Moreover, when second- and third-generation of ALK inhibitors are available on the market; new models need to be updated using these ALK inhibitors. Finally, some model variables were derived or obtained from China, while some information was from global or regional clinical trials not just Chinese participants. However, the various sensitivity analyses could help examine these uncertainties.

In conclusion, the gene-guided (NGS and multiplex PCR) crizotinib therapy is cost-effective compared to standard chemotherapy without ALK testing, when the PAP of crizotinib is available.
